# Rapid Detection of *Bifidobacterium bifidum* in Feces Sample by Highly Sensitive Quartz Crystal Microbalance Immunosensor

**DOI:** 10.3389/fchem.2020.00548

**Published:** 2020-07-07

**Authors:** Kaijian Hou, Pingsen Zhao, Yongru Chen, Guiping Li, Yu Lin, Danjie Chen, Dan Zhu, Zezhen Wu, Danchun Lian, Xiaojun Huang, Jilin Li

**Affiliations:** ^1^Department of Endocrine and Metabolic Diseases, Longhu Hospital, The First Affiliated Hospital of Shantou University Medical College, Shantou, China; ^2^Department of Laboratory Medicine, Yuebei People's Hospital, Shantou University Medical College, Shaoguan, China; ^3^Department of Emergency Intensive Care Unit (EICU), The First Affiliated Hospital of Shantou University Medical College, Shantou, China; ^4^Department of Endocrine and Metabolic Diseases, The Third People's Hospital of Huizhou, Huizhou, China; ^5^Department of Endocrinology, Jieyang People's Hospital, Jieyang, China; ^6^Department of Endocrinology, Puning People's Hospital, Puning, China; ^7^Department of Cardiology, The Second Affiliated Hospital of Shantou University Medical College, Shantou, China

**Keywords:** quartz crystal microbalance sensor, *Bifidobacterium bifidum*, immunosensor, Au nanoparticle, feces sample

## Abstract

In this work, a quartz crystal microbalance (QCM) sensor has been fabricated using immunoassay for sensitive determination of *Bifidobacterium bifidum*. Au nanoparticle has been used for amplifying sandwich assays. The proposed immunosensor exhibited a linear detection range between 10^3^ and 10^5^ CFU/mL with a limit of detection of 2.1 × 10^2^ CFU/mL. The proposed immunosensor exhibited good selectivity for *B. bifidum* sensing with low cross reactivity for other foodborne pathogens such as *Lactobacillus acidophilus, Listeria monocytogenes*, and *Escherichia coli*. In addition, the proposed immunosensor has been successfully used for *B. bifidum* detection in feces samples and food samples. The frequency decreases of 12, 17, and 10 Hz were observed from the milk samples consisting of the mixtures of *L. acidophilus, L. monocytogenes*, and *E. coli*. The frequency decreases of 8, 15, and 7 Hz were observed from the feces samples consisting of the mixtures of *L. acidophilus, L. monocytogenes*, and *E. coli*.

## Introduction

Under normal circumstances, the intestinal microorganisms in the human body form a relatively balanced state. Once the balance is damaged, it will lead to the imbalance of intestinal flora (Horie et al., 2017; Xue et al., 2018). Some intestinal microorganisms, such as *Clostridium perfringens*, overproduce in the intestinal tract and produce harmful substances such as ammonia, amines, hydrogen sulfide, manure, indole, nitrite, and bacterial toxins, which will further affect the health of the body (Ashida et al., 2018; Duranti et al., 2019). *Bifidobacterium bifidum* is an important intestinal beneficial microorganism. *B. bifidum*, as a physiological beneficial bacterium, has many important physiological functions for human health, such as biological barrier, anti-tumor, immune enhancement, improvement of gastrointestinal function, and anti-aging (Wang et al., 2016; Mauras et al., 2018; Din et al., 2020). *B. bifidum* can inhibit the growth of harmful bacteria, resist the infection of pathogenic bacteria, and synthesize vitamins needed by human body. It can promote the absorption of minerals, produce organic acids such as acetic acid, propionic acid, butyric acid, and lactic acid to stimulate intestinal peristalsis and promote defecation. It can prevent constipation, purify the intestinal environment, decompose carcinogens, stimulate the human immune system, and improve the disease resistance (Gomi et al., 2018; Bondue et al., 2019; Speciale et al., 2019). It is of crucial importance to identify *B. bifidum* either in food and excreta. Although the normal microbiological isolation methods have been widely used for identifying bacterial strains, they are time-consuming (Yang et al., 2017; Cheng et al., 2018; Faraki et al., 2020). Therefore, the development of a fast method for *B. bifidum* identification is very essential for sample scanning.

The biosensor first immobilizes the bioactive components (enzyme, antibody, tissue, cell) on the transducer. When the target analyte is recognized by the immobilized bioactive components, the biochemical reaction can be immediately converted into a quantifiable electrical signal through the transducer (Fu et al., 2015; Huertas et al., 2019; Alamgholiloo et al., 2020; Fouladgar et al., 2020; Karimi-Maleh et al., 2020). Since the rise of biosensor in the late 1960s, after nearly half a century of development, biosensor has become a comprehensive and interdisciplinary field, which is used in food safety testing, environmental testing, and clinical diagnosis. QCM is a new type of micro mass sensor based on quartz crystal resonance, which was developed in 1960s. According to the piezoelectric effect of quartz crystal, the resonance frequency of quartz crystal will change with the mass of adsorbed material, and they are in a positive proportion (Bearzotti et al., 2017; Speller et al., 2017; Wang A. et al., 2017; Ayankojo et al., 2018). QCM immunosensor is a specific biosensor combining the high sensitivity of quartz crystal and the high specificity of immune response (Bearzotti et al., 2017; Speller et al., 2017; Wang L. et al., 2017; Ayankojo et al., 2018; Zhang et al., 2018). At present, QCM immunosensor has been widely used in clinical testing, food hygiene, environmental testing, as well as other fields of chemical analysis, and biological analysis. The detection of bacteria by QCM is a new and attractive Research Topic in electronic informatics and medicine (Muckley et al., 2016; Tai et al., 2016; Chen et al., 2018; Ding et al., 2018; Lal and Tiwari, 2018; Temel et al., 2019).

In this work, a QCM immunosensor has been developed for sensitive determination of *B. bifidum*. Monoclonal and polyclonal antibody have been used with the signal amplifying based on the Au nanoparticle. The proposed immunosensor exhibited a wide linear detection range with a low detection of limit. The sensitivity was enhanced when the antibody-conjugated Au nanoparticle. We believe the proposed assay was validated by cross reactivity investigation using *Lactobacillus acidophilus, Listeria monocytogenes* and *Escherichia coli*.

## Materials and Methods

### Reagents, Bacterial Strains, and Instruments

Polyclonal antibody and mouse monoclonal antibody against *B. bifidum* were purchased from San Ying Biotechnology Co., Ltd (Wuhan, China). Mouse IgG, 11-mercaptoundecanoic acid (11-MUDA), bovine serum albumin (BSA), and ethanolamine hydrochloride were purchased from Linc-Bio Science Co., Ltd (Shanghai, China). Au colloidal (AuNPs) with 20 nm was purchased from Shenzhen Nano Tech Co., Ltd (Shenzhen, China). All other common regents were supplied by Sinopharm Chemical Reagent Co., Ltd (Shanghai, China) and used without further purification. A QCA922 quartz crystal analyzer (Princeton, USA) has been used for sensing with an Au coated chips. *Bifidobacterium bifidum* (*B. bifidum*), *L. acidophilus, L. monocytogenes*, and *E. coli* strains were purchased from American Type Culture Collection (ATCC, Manassas, VA). MRS (de Man, Rogosa, and Sharpe) and Bifidus Selective Medium (BSM) were used as growth media.

### Sensor Chip Preparation

Piranha solution has been used for removing any impurities on the QCM Au chip. Then, the Au chip was immersed into 20 mM thiol (11-MUDA, prepared using ethanol) overnight for fabricating carboxy-terminated thiol layer on the Au surface. After rinse by ethanol and water, the Au chip was used for baseline correction under 10 mM PBS (pH 7.4). A mixture solution of EDC (0.4 M)-NHS (0.1 M) at ratio of 1:1 was then used for sensor activation. Then, different concentrations of polyclonal or mouse monoclonal antibodies against *B. bifidum* were immobilized on the Au chip. Then, capture and control were carried out by injected either polyclonal or mouse monoclonal antibodies and mouse IgG antibody. Then, BSA (50 μg/mL) and ethanolamine (1 M) were used for blocking and capping the sensors.

### Detection of *B. bifidum*

Direct detection of *B. bifidum* was carried out by the injection of different concentrations of *B. bifidum* cells suspension prepared in PBS over above-mentioned polyclonal, mouse monoclonal, and mouse IgG antibodies fabricated sensor. The detection was carried out by comparing either *B. bifidum* captured polyclonal or mouse monoclonal antibodies with the control group.

The sandwich assay with antibody-AuNPs conjugation was prepared according to previous methods with some modifications (Uludag and Tothill, 2012). Typically, 0.1 mL of polyclonal or mouse monoclonal antibodies was added into 0.5 mL of AuNPs suspension with 2 h slow stirring. Then, 0.1 mL of BSA (10%) was introduced to the mixture with 2 h slow stirring. A centrifugation process was carried out to remove the excess of BSA and AuNPs. The detection process of using antibody-AuNPs conjugation was similar to the direct detection except the polyclonal and mouse monoclonal antibodies were replaced by the polyclonal-AuNPs and mouse monoclonal-AuNPs antibodies. Non-linear regression with four parameter logistic equations has been used for construing the calibration curves (Karpinski, 1990).

### Selectivity Test

*L. acidophilus, L. monocytogenes*, and *E. coli* have been used for evaluating the specificity of the assay to *B. bifidum*. The selectivity test only carried out using antibody-AuNPs conjugation. The detection process of was similar to the above-mentioned protocol except replaced *B. bifidum* by *L. acidophilus, L. monocytogenes*, and *E. coli*.

### Real Sample Preparation

The sensing of *B. bifidum* in milk and feces were investigated. Fresh milk was purchased form local supermarket. Feces samples were provided by The First Affiliated Hospital of Shantou University Medical College. For the sensing of *B. bifidum*, 1 g of real sample was dispersed using 50 mL PBS and transferred into a filtering stomacher bag. Then, 0.5 mL of the *B. bifidum* suspension was inoculated into both real samples at 37°C for 2 h. All liquid samples after preparation were directly used for sensing purpose.

## Results and Discussion

**Figure 1:**
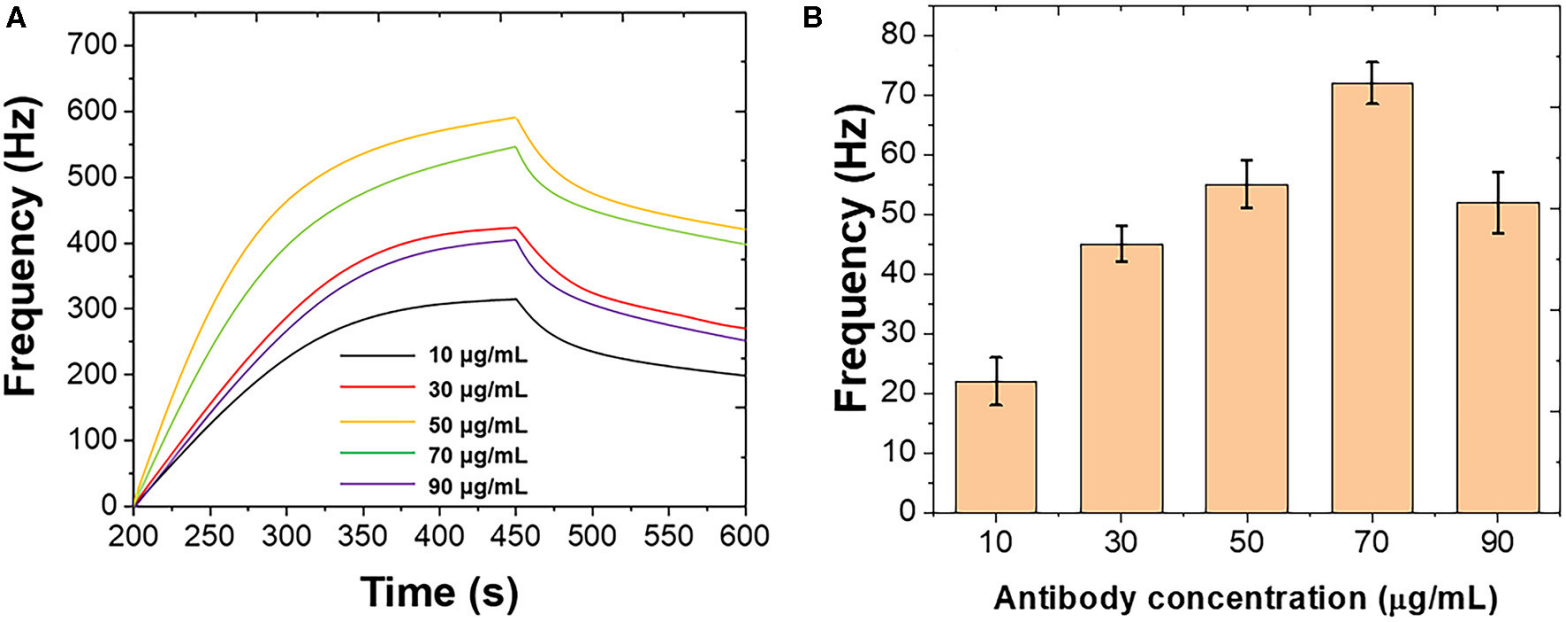
**(A)** Frequencies recorded at QCM when different concentrations of polyclonal antibody immobilized on the Au chip surface. **(B)** Frequencies performance of QCM after introduction of *B. bifidum* after the immobilization of different concentrations of polyclonal antibody.

Figure 1 shows the optimization of the immobilization of polyclonal antibody on the Au chip surface. Different concentrations of polyclonal antibody were introduced using the standard *B. bifidum*. As shown in Figure 1A, the frequency was initially increased when the polyclonal antibody increased from 10 to 50 μg/mL. Then, the frequency decreased when the further increase of the polyclonal antibody. Figure 1B shows the binding response of *B. bifidum* at these concentrations of polyclonal antibody used. It can be seen that the best response was achieved when the concentration of polyclonal antibody at 50 μg/mL. Therefore, 50 μg/mL of polyclonal antibody has been selected in this study.

**Figure 2:**
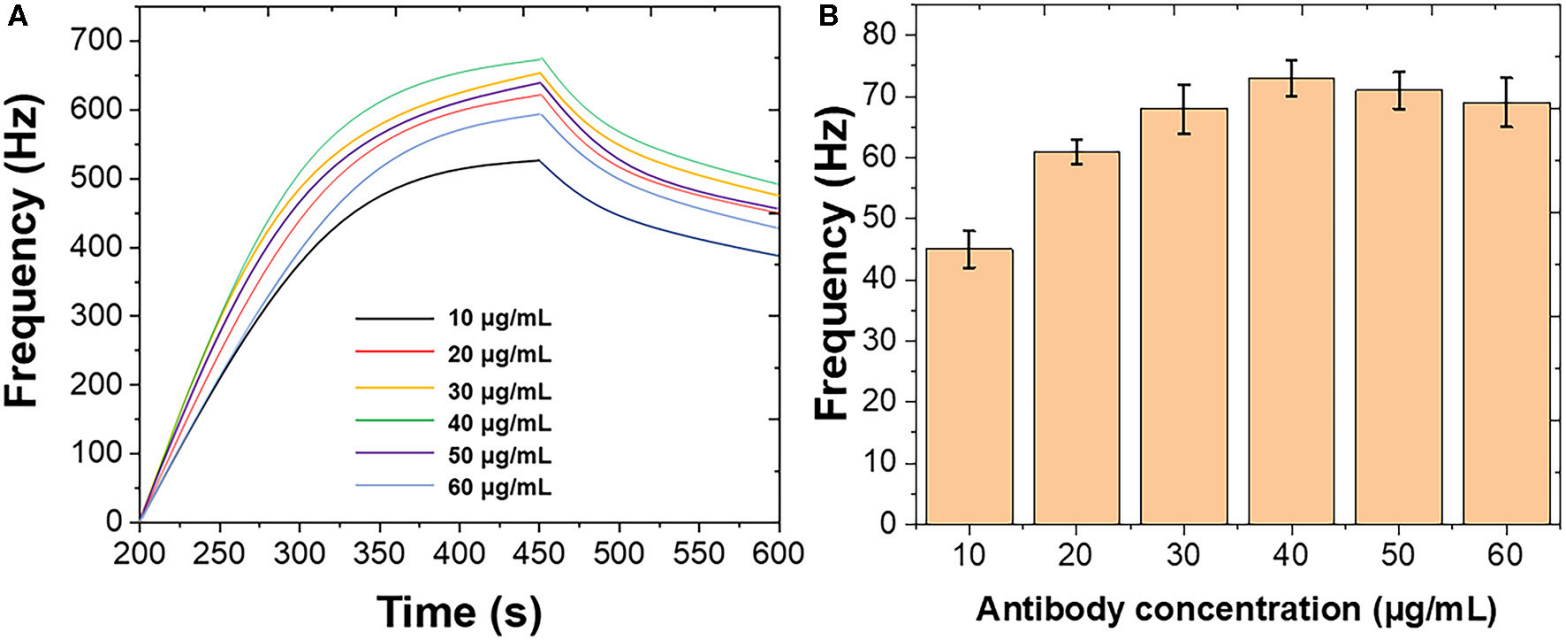
**(A)** Frequencies recorded at QCM when different concentrations of mouse monoclonal antibody immobilized on the Au chip surface. **(B)** Frequencies performance of QCM after introduction of *B. bifidum* after the immobilization of different concentrations of mouse monoclonal antibody.

Figure 2 shows the optimization of the immobilization of mouse monoclonal antibody on the Au chip surface. Different concentrations of mouse monoclonal antibody were introduced using the standard *B. bifidum*. As shown in Figure 2A, the frequency was initially increased when the mouse monoclonal antibody increased from 10 to 40 μg/mL. Then, the frequency decreased when the further increase of the mouse monoclonal antibody. Figure 2B shows the binding response of *B. bifidum* at these concentrations of mouse monoclonal antibody used. It can be seen that the best response was achieved when the concentration of mouse monoclonal antibody at 50 μg/mL. Therefore, 50 μg/mL of mouse monoclonal antibody has been selected in this study. Both studies suggested a high concentration of antibodies could result in a high steric hindrance, which lower the sensitivity of the sensor. Similar results were obtained for *Campylobacter jejuni* and *Vibrio harveyi* as well (Buchatip et al., 2010; Masdor et al., 2016).

**Figure 3:**
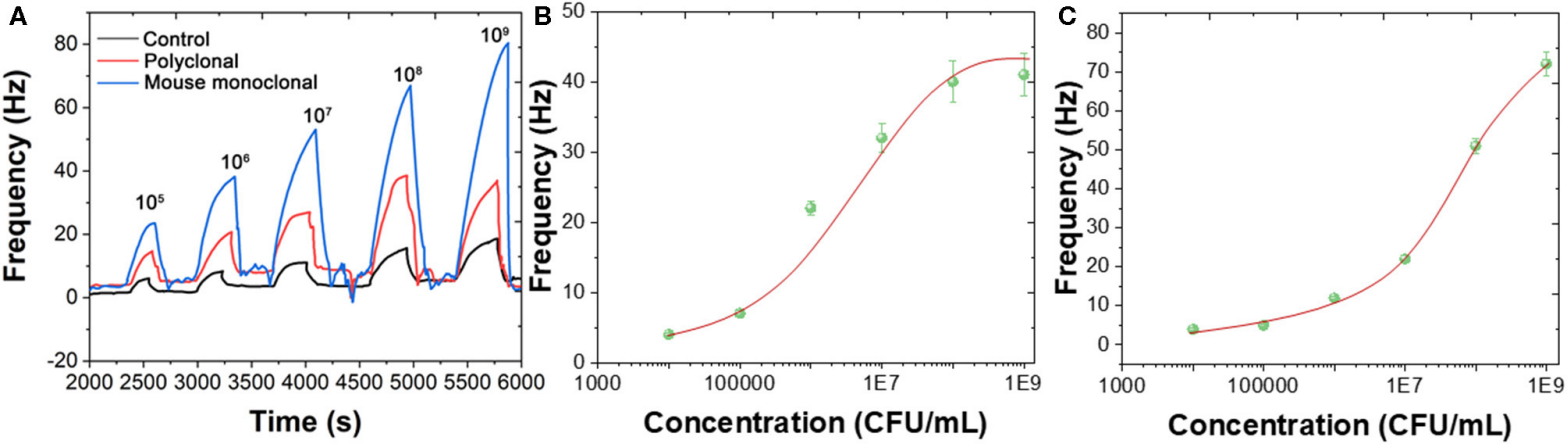
**(A)** Frequencies recorded at QCM when different concentrations of *B. bifidum* introduced using polyclonal antibody immobilized sensor, mouse monoclonal antibody immobilized sensor, and mouse IgG antibody immobilized sensor. Plots of Frequency vs. concentrations of *B. bifidum* using **(B)** polyclonal antibody immobilized sensor and **(C)** mouse monoclonal antibody immobilized sensor.

*B. bifidum* can be directed detected using both polyclonal and mouse monoclonal antibodies immobilized Au chips. Figure 3 shows the frequency changes when different concentrations of *B. bifidum* introduced in the QCM system in the presence of polyclonal antibody immobilized sensor, mouse monoclonal antibody immobilized sensor, and mouse IgG antibody immobilized sensor. As shown in Figures 3B,C, sigmoidal relationship between frequency change signals and concentrations of *B. bifidum* were obtained for both polyclonal antibody immobilized sensor and mouse monoclonal antibody immobilized sensor. The linear portions of the concentrations of *B. bifidum* between 10^5^-10^7^ and 10^6^-10^8^ CFU/mL were obtained for polyclonal antibody immobilized sensor and mouse monoclonal antibody immobilized sensor, respectively. The limit of detection (LOD) of polyclonal antibody immobilized sensor and mouse monoclonal antibody immobilized sensor can be calculated to be 3.0 × 10^5^ and 3.0 × 10^6^ CFU/mL, respectively. Since the polyclonal antibody immobilized sensor exhibited a lower LOD, it has been further used for construction of sandwich assay.

**Figure 4:**
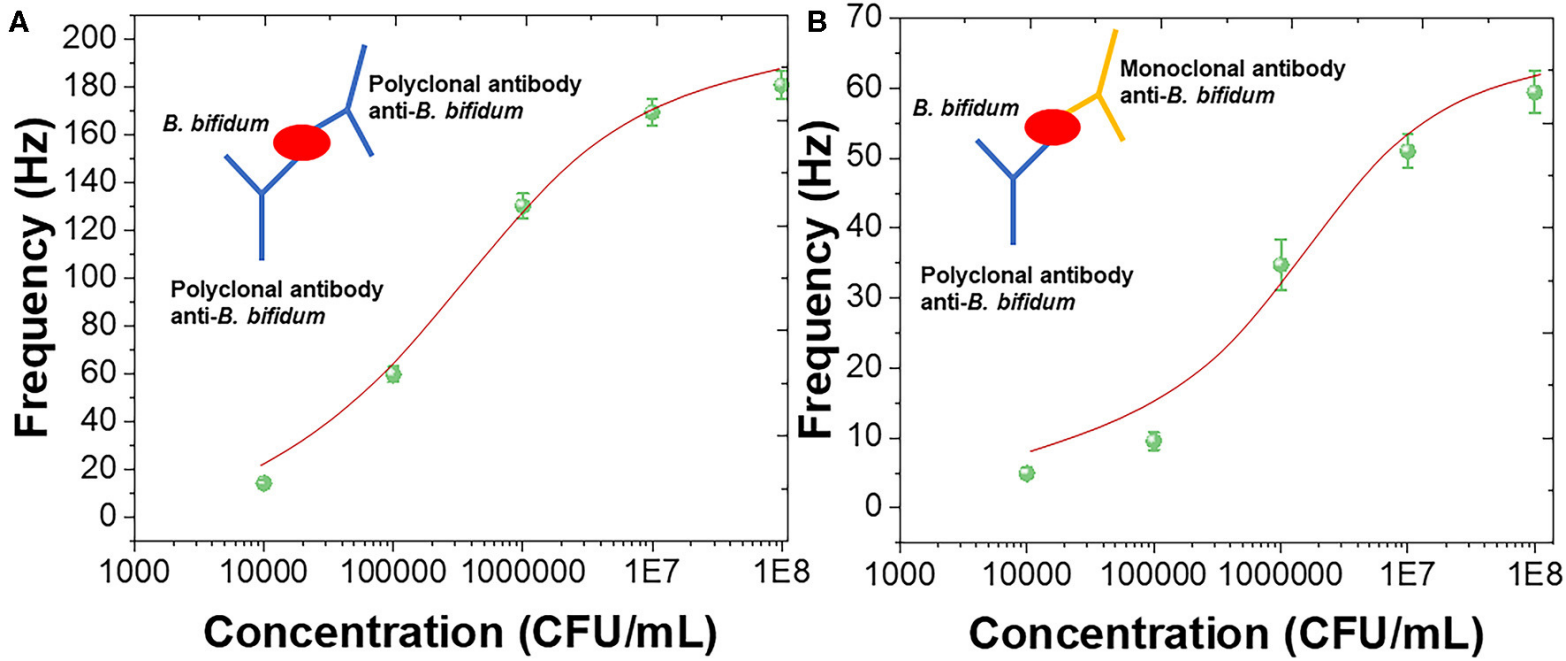
Frequency vs. concentrations of *B. bifidum* using **(A)** polyclonal antibody and **(B)** mouse monoclonal antibody as the detection antibodies.

The sandwich assay has been constructed using polyclonal antibody against *B. bifidum*. The sandwich assay could improve sensitivity as well as prevented the false negative results (Amani et al., 2018). Figure 4A shows the sensing performance of using polyclonal antibody as the capture and detection antibody with schematic diagram. Figure 4B shows the sensing performance of using polyclonal antibody as the capture and mouse monoclonal antibody as the detection antibody with schematic diagram. It can be seen that both sandwich assay exhibited sigmoidal relationship between frequency and concentrations of *B. bifidum*. Linear portions of the concentrations of *B. bifidum* between 10^4^-10^8^ and 10^5^-10^8^ CFU/mL were observed using polyclonal antibody and mouse monoclonal antibody as the detection antibodies, respectively. The LOD of using polyclonal antibody and mouse monoclonal antibody as the detection antibodies can be calculated to be 2.0 × 10^4^ and 3.3 × 10^5^ CFU/mL, respectively. These results indicated that the sandwich assay could increase the sensitivity of the QCM sensor (Cervera-Chiner et al., 2018; Makhneva et al., 2018; Pohanka, 2020).

**Figure 5:**
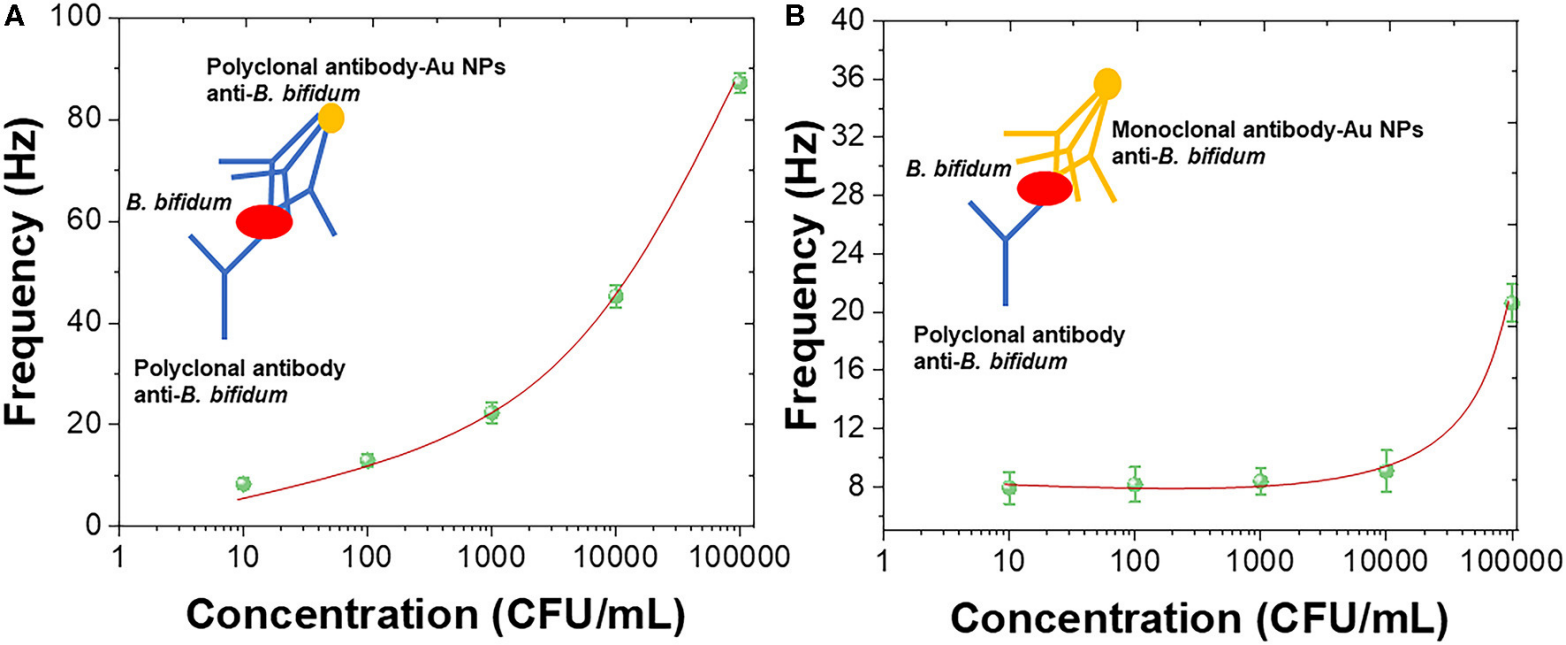
Frequency vs. concentrations of *B. bifidum* using **(A)** polyclonal antibody-AuNPs conjugation and **(B)** mouse monoclonal antibody-AuNPs conjugation as the detection layers.

In order to further enhance the sensitivity of the QCM immunosensor, Au nanoparticles were introduced for forming the antibody-AuNPs conjugation (Zheng et al., 2015; Wang L. et al., 2017; Fu et al., 2020; Xu et al., 2020). Figure 5A shows the sensing performance of using polyclonal antibody-AuNPs conjugation as detection layer with schematic diagram. Figure 5B shows the sensing performance of using mouse monoclonal antibody-AuNPs conjugation as detection layer with schematic diagram. A linear portions of the concentrations of *B. bifidum* between 10^3^ and 10^5^ CFU/mL was observed using polyclonal antibody-AuNPs conjugation as detection layer. The LOD of using mouse monoclonal antibody-AuNPs conjugation can be calculated to be 2.1 × 10^2^ CFU/mL. No clear linear portions of the concentrations of *B. bifidum* can be observed using mouse monoclonal antibody-AuNPs conjugation as detection layer. The results recorded in this study are very competitive with previous reports (Fung and Wong, 2001; Guo et al., 2012; Skládal, 2016; Della Ventura et al., 2017; Wang R. et al., 2017; Gao et al., 2018; Pohanka, 2018a,b, 2019). Table 1 showed the sensing performance comparison.

**Table 1:** Sensing performance of the proposed QCM sensor with other reports.

**Analyte**	**Linear detection range**	**Limit of detection**	**References**
*S. paratyphi*	10^2^-10^5^ CFU/mL	1.7 × 10^2^ CFU/mL	Fung and Wong, 2001
*E. coli* O157:H7	0–1 log CFU/mL	–	Guo et al., 2012
α-Amylase	–	1 μg/mL	Della Ventura et al., 2017
H5N1 avian influenza virus	2^−4^-2^4^ HAUs/50 μL	2^−4^ HAU/50 μL	Wang L. et al., 2017
Albumin	35–55 mg/mL	0.234 mg/mL	Pohanka, 2018a
*S. aureus*	–	5.18 × 10^8^ CFU/mL	Pohanka, 2020
*B. bifidum*	10^3^-10^5^ CFU/mL	2.1 × 10^2^ CFU/mL	This work

**Figure 6:**
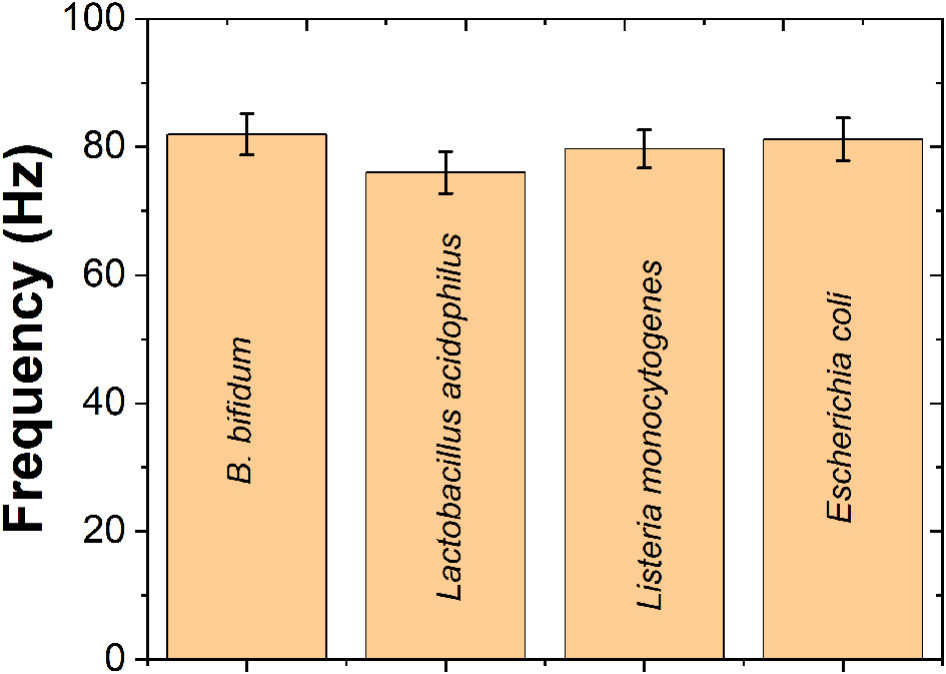
Selectivity performance of the proposed QCM immunosensor toward *B. bifidum* sensing compared with that of the *Lactobacillus acidophilus, Listeria monocytogenes* and *Escherichia coli*.

The selectivity of the proposed QCM immunosensor has been tested by *L. acidophilus, L. monocytogenes*, and *E. coli*. As shown in Figure 6, <10% of cross reactivities were observed by *L. acidophilus, L. monocytogenes*, and *E. coli*, suggesting the proposed QCM immunosensor exhibited an excellent selectivity toward *B. bifidum*.

**Figure 7:**
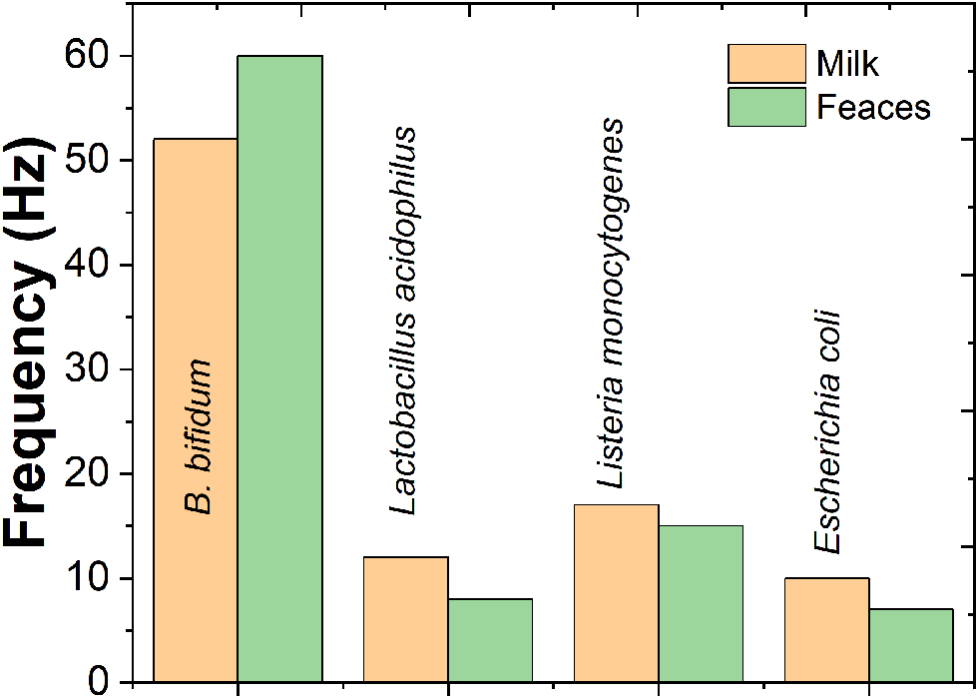
Specific detection of 10^4^ CFU/mL of *B. bifidum, Lactobacillus acidophilus, Listeria monocytogenes* and *Escherichia coli* inoculated on milk and feces samples.

The applicability of the novel QCM immunosensor to determine *B. bifidum* in milk and feces were also carried out. For the detection of *B. bifidum* in real samples, the QCM sensor was firstly calibrated by the measurement in the absence of *B. bifidum*. As shown in Figure 7, frequency change of 3 and 7 Hz were recorded from the milk sample and feces sample containing 10^4^ CFU/mL of *B. bifidum*, respectively. These results suggested the proposed QCM immunosensor could be used for real sample test. In addition, the frequency decreases of 12, 17, and 10 Hz were observed from the milk samples consisting of the mixtures of *L. acidophilus, L. monocytogenes*, and *E. coli*. The frequency decreases of 8, 15, and 7 Hz were observed from the feces samples consisting of the mixtures of *L. acidophilus, L. monocytogenes*, and *E. coli*. The results indicate that the QCM system developed in the present study is practical for the simultaneous enrichment and detection of viable *B. bifidum* in real samples.

## Conclusion

In this work, an advanced piezoelectric biosensor-QCM system was proposed. In order to further enhance the sensitivity of the QCM immunosensor, Au nanoparticles were introduced for forming the antibody-AuNPs conjugation. A linear portions of the concentrations of *B. bifidum* between 10^3^ and 10^5^ CFU/mL was observed using polyclonal antibody-AuNPs conjugation as detection layer. The LOD of using mouse monoclonal antibody-AuNPs conjugation can be calculated to be 2.1 × 10^2^ CFU/mL. The selectivity of the proposed QCM immunosensor has been tested by *L. acidophilus, L. monocytogenes*, and *E. coli*. The applicability of the novel QCM immunosensor to determine *B. bifidum* in milk and feces were tested. The frequency decreases of 12, 17, and 10 Hz were observed from the milk samples consisting of the mixtures of *L. acidophilus, L. monocytogenes*, and *E. coli*. The frequency decreases of 8, 15, and 7 Hz were observed from the feces samples consisting of the mixtures of *L. acidophilus, L. monocytogenes*, and *E. coli*.

## Data Availability Statement

The raw data supporting the conclusions of this article will be made available by the authors, without undue reservation.

## Ethics Statement

Ethical review and approval was not required for the study on human participants in accordance with the local legislation and institutional requirements. Written informed consent from the participants was not required to participate in this study in accordance with the national legislation and the institutional requirements.

## Author Contributions

KH, YC, and JL contributed conception and design of the study. GL, YL, and DC conducted QCM experiments. DZ and ZW performed the statistical analysis. DL and XH did the characterizations. KH and YC wrote the manuscript. All authors contributed to the article and approved the submitted version.

## Conflict of Interest

The authors declare that the research was conducted in the absence of any commercial or financial relationships that could be construed as a potential conflict of interest.

## References

[B1] Alamgholiloo H. Rostamnia S. Hassankhani A. Liu X. Eftekhari A. Hasanzadeh A.. (2020). Formation and stabilization of colloidal ultra-small palladium nanoparticles on diamine-modified Cr-MIL-101: synergic boost to hydrogen production from formic acid. J. Colloid Interface Sci. 567, 126–135. 10.1016/j.jcis.2020.01.087 32044541

[B2] Amani J. Maleki M. Khoshroo A. Sobhani-Nasab A. Rahimi-Nasrabadi M. (2018). An electrochemical immunosensor based on poly p-phenylenediamine and graphene nanocomposite for detection of neuron-specific enolase via electrochemically amplified detection. Anal. Biochem. 548, 53–59. 10.1016/j.ab.2018.02.024 29486202

[B3] Ashida H. Tanigawa K. Kiyohara M. Katoh T. Katayama T. Yamamoto K. (2018). Bifunctional properties and characterization of a novel sialidase with esterase activity from *Bifidobacterium bifidum*. Biosci. Biotechnol. Biochem. 82, 2030–2039. 10.1080/09168451.2018.1497944 30027820

[B4] Ayankojo A. G. Reut J. Boroznjak R. Öpik A. Syritski V. (2018). Molecularly imprinted poly (meta-phenylenediamine) based QCM sensor for detecting Amoxicillin. Sens. Actuators B Chem. 258, 766–774. 10.1016/j.snb.2017.11.194 PMC8626155 34866797

[B5] Bearzotti A. Macagnano A. Papa P. Venditti I. Zampetti E. (2017). A study of a QCM sensor based on pentacene for the detection of BTX vapors in air. Sens. Actuators B Chem. 240, 1160–1164. 10.1016/j.snb.2016.09.097

[B6] Bondue P. Crèvecoeur S. Brose F. Daube G. Seghaye M.-C. Griffiths M. W.. (2019). Corrigendum: cell-free spent media obtained from *bifidobacterium bifidum* and Bifidobacterium crudilactis grown in media supplemented with 3′-Sialyllactose modulate virulence gene expression in *Escherichia coli* O157: H7 and *Salmonella Typhimurium*. Front. Microbiol. 10:2490. 10.3389/fmicb.2019.02490 31798537 PMC6868515

[B7] Buchatip S. Ananthanawat C. Sithigorngul P. Sangvanich P. Rengpipat S. Hoven V. P. (2010). Detection of the shrimp pathogenic bacteria, Vibrio harveyi, by a quartz crystal microbalance-specific antibody based sensor. Sens. Actuators B Chem. 145, 259–264. 10.1016/j.snb.2009.12.003

[B8] Cervera-Chiner L. Juan-Borrás M. March C. Arnau A. Escriche I. Montoya Á.. (2018). High fundamental frequency quartz crystal microbalance (HFF-QCM) immunosensor for pesticide detection in honey. Food Control 92, 1–6. 10.1016/j.foodcont.2018.04.026 31965575

[B9] Chen W. Deng F. Xu M. Wang J. Wei Z. Wang Y. (2018). GO/Cu2O nanocomposite based QCM gas sensor for trimethylamine detection under low concentrations. Sens. Actuators B Chem. 273, 498–504. 10.1016/j.snb.2018.06.062

[B10] Cheng R. Yao J. Wan Q. Guo J. Pu F. Shi L.. (2018). Oral administration of *Bifidobacterium bifidum* TMC3115 to neonatal mice may alleviate IgE-mediated allergic risk in adulthood. Benef. Microbes 9, 815–828. 10.3920/BM2018.0005 29888657

[B11] Della Ventura B. Sakač N. Funari R. Velotta R. (2017). Flexible immunosensor for the detection of salivary α-amylase in body fluids. Talanta 174, 52–58. 10.1016/j.talanta.2017.05.075 28738617

[B12] Din A. U. Hassan A. Zhu Y. Zhang K. Wang Y. Li T.. (2020). Inhibitory effect of *Bifidobacterium bifidum* ATCC 29521 on colitis and its mechanism. J. Nutr. Biochem. 79:108353. 10.1016/j.jnutbio.2020.108353 32145470

[B13] Ding X. Chen X. Chen X. Zhao X. Li N. (2018). A QCM humidity sensor based on fullerene/graphene oxide nanocomposites with high quality factor. Sens. Actuators B Chem. 266, 534–542. 10.1016/j.snb.2018.03.143

[B14] Duranti S. Lugli G. A. Milani C. James K. Mancabelli L. Turroni F.. (2019). *Bifidobacterium bifidum* and the infant gut microbiota: an intriguing case of microbe-host co-evolution. Environ. Microbiol. 21, 3683–3695. 10.1111/1462-2920.14705 31172651

[B15] Faraki A. Noori N. Gandomi H. Banuree S. A. H. Rahmani F. (2020). Effect of Auricularia auricula aqueous extract on survival of *Lactobacillus acidophilus* La-5 and *Bifidobacterium bifidum* Bb-12 and on sensorial and functional properties of synbiotic yogurt. Food Sci. Nutr. 8, 1254–1263. 10.1002/fsn3.1414 32148831 PMC7020330

[B16] Fouladgar M. Karimi-Maleh H. Opoku F. Govender P. P. (2020). Electrochemical anticancer drug sensor for determination of raloxifene in the presence of tamoxifen using graphene-CuO-polypyrrole nanocomposite structure modified pencil graphite electrode: theoretical and experimental investigation. J. Mol. Liq. 311:113314 10.1016/j.molliq.2020.113314

[B17] Fu L. Yu S. Thompson L. Yu A. (2015). Development of a novel nitrite electrochemical sensor by stepwise in situ formation of palladium and reduced graphene oxide nanocomposites. RSC Adv. 5, 40111–40116. 10.1039/C5RA02661J

[B18] Fu L. Zheng Y. Zhang P. Zhang H. Xu Y. Zhou J.. (2020). Development of an electrochemical biosensor for phylogenetic analysis of Amaryllidaceae based on the enhanced electrochemical fingerprint recorded from plant tissue. Biosens. Bioelectron. 159:112212. 10.1016/j.bios.2020.112212 32364933

[B19] Fung Y. Wong Y. (2001). Self-assembled monolayers as the coating in a quartz piezoelectric crystal immunosensor to detect Salmonella in aqueous solution. Anal. Chem. 73, 5302–5309. 10.1021/ac010655y 11721933

[B20] Gao K. Cui S. Liu S. (2018). Development of an electrochemical quartz crystal microbalance-based immunosensor for C-reactive protein determination. Int. J. Electrochem. Sci. 13, 812–821. 10.20964/2018.01.49

[B21] Gomi A. Yamaji K. Watanabe O. Yoshioka M. Miyazaki K. Iwama Y.. (2018). *Bifidobacterium bifidum* YIT 10347 fermented milk exerts beneficial effects on gastrointestinal discomfort and symptoms in healthy adults: a double-blind, randomized, placebo-controlled study. J. Dairy Sci. 101, 4830–4841. 10.3168/jds.2017-13803 29573807

[B22] Guo X. Lin C.-S. Chen S.-H. Ye R. Wu V. C. (2012). A piezoelectric immunosensor for specific capture and enrichment of viable pathogens by quartz crystal microbalance sensor, followed by detection with antibody-functionalized gold nanoparticles. Biosens. Bioelectron. 38, 177–183. 10.1016/j.bios.2012.05.024 22683250

[B23] Horie M. Miura T. Hirakata S. Hosoyama A. Sugino S. Umeno A.. (2017). Comparative analysis of the intestinal flora in type 2 diabetes and nondiabetic mice. Exp. Anim. 66, 405–416. 10.1538/expanim.17-0021 28701620 PMC5682353

[B24] Huertas C. S. Calvo Lozano O. Mitchell A. Lechuga L. M. (2019). Advanced evanescent-wave optical biosensors for the detection of nucleic acids: an analytic perspective. Front. Chem. 7:724. 10.3389/fchem.2019.00724 31709240 PMC6823211

[B25] Karimi-Maleh H. Karimi F. Alizadeh M. Sanati A. L. (2020). Electrochemical sensors, a bright future in the fabrication of portable kits in analytical systems. Chem. Rec. [Epub ahead of print]. 10.1002/tcr.201900092 31845511

[B26] Karpinski K. (1990). Optimality assessment in the enzyme-linked immunosorbent assay (ELISA). Biometrics 46, 381–390. 10.2307/2531443 2364128

[B27] Lal G. Tiwari D. (2018). Investigation of nanoclay doped polymeric composites on piezoelectric Quartz Crystal Microbalance (QCM) sensor. Sens. Actuators B Chem. 262, 64–69. 10.1016/j.snb.2018.01.200

[B28] Makhneva E. Farka Z. Skládal P. Zajíčková L. (2018). Cyclopropylamine plasma polymer surfaces for label-free SPR and QCM immunosensing of Salmonella. Sens. Actuators B Chem. 276, 447–455. 10.1016/j.snb.2018.08.055

[B29] Masdor N. A. Altintas Z. Tothill I. E. (2016). Sensitive detection of Campylobacter jejuni using nanoparticles enhanced QCM sensor. Biosens. Bioelectron. 78, 328–336. 10.1016/j.bios.2015.11.033 26649490

[B30] Mauras A. Chain F. Faucheux A. Ruffié P. Gontier S. Ryffel B.. (2018). A new Bifidobacteria expression system (BEST) to produce and deliver interleukin-10 in *Bifidobacterium bifidum*. Front. Microbiol. 9:3075. 10.3389/fmicb.2018.03075 30622516 PMC6308194

[B31] Muckley E. S. Lynch J. Kumar R. Sumpter B. Ivanov I. N. (2016). PEDOT: PSS/QCM-based multimodal humidity and pressure sensor. Sens. Actuators B Chem. 236, 91–98. 10.1016/j.snb.2016.05.054

[B32] Pohanka M. (2018a). Piezoelectric immunosensor for the determination of immunoglobulin G. Int. J. Electrochem. Sci. 13, 8784–8791. 10.20964/2018.09.01

[B33] Pohanka M. (2018b). The determination of human albumin by a quartz crystal microbalance immunosensor. Int. J. Electrochem. Sci. 13, 8471–8480. 10.20964/2018.09.14

[B34] Pohanka M. (2019). Piezoelectric immunosensor for the determination of c-reactive protein. Int. J. Electrochem. Sci. 14, 8470–8478. 10.20964/2019.09.02

[B35] Pohanka M. (2020). QCM immunosensor for the determination of *Staphylococcus aureus* antigen. Chem. Pap. 74, 451–458. 10.1007/s11696-019-00889-5

[B36] Skládal P. (2016). Piezoelectric biosensors. TrAC Trends Anal. Chem. 79, 127–133. 10.1016/j.trac.2015.12.00918080881

[B37] Speciale I. Verma R. Di Lorenzo F. Molinaro A. Im S.-H. de Castro C. (2019). *Bifidobacterium bifidum* presents on the cell surface a complex mixture of glucans and galactans with different immunological properties. Carbohydr. Polym. 218, 269–278. 10.1016/j.carbpol.2019.05.006 31221330

[B38] Speller N. C. Siraj N. McCarter K. S. Vaughan S. Warner I. M. (2017). QCM virtual sensor array: vapor identification and molecular weight approximation. Sens. Actuators B Chem. 246, 952–960. 10.1016/j.snb.2017.02.042

[B39] Tai H. Zhen Y. Liu C. Ye Z. Xie G. Du X. (2016). Facile development of high performance QCM humidity sensor based on protonated polyethylenimine-graphene oxide nanocomposite thin film. Sens. Actuators B Chem. 230, 501–509. 10.1016/j.snb.2016.01.105

[B40] Temel F. Erdemir S. Ozcelik E. Tabakci B. Tabakci M. (2019). Rapid and real-time detection of arginine enantiomers by QCM sensor having a Calix [4] arene receptor bearing asymmetric centers. Talanta 204, 172–181. 10.1016/j.talanta.2019.05.093 31357279

[B41] Uludag Y. Tothill I. E. (2012). Cancer biomarker detection in serum samples using surface plasmon resonance and quartz crystal microbalance sensors with nanoparticle signal amplification. Anal. Chem. 84, 5898–5904. 10.1021/ac300278p 22681722

[B42] Wang A. Wang C. Fu L. Wong-Ng W. Lan Y. (2017). Recent advances of graphitic carbon nitride-based structures and applications in catalyst, sensing, imaging, and LEDs. Nano-Micro Lett. 9:47. 10.1007/s40820-017-0148-2 30393742 PMC6199047

[B43] Wang G.-H. Chen C.-Y. Lin C.-P. Huang C.-L. Lin C. Cheng C.-Y. (2016). Tyrosinase inhibitory and antioxidant activities of three *Bifidobacterium bifidum*-fermented herb extracts. Ind. Crops Prod. 89, 376–382. 10.1016/j.indcrop.2016.05.037

[B44] Wang L. Wang Z. Xiang Q. Chen Y. Duan Z. Xu J. (2017). High performance formaldehyde detection based on a novel copper (II) complex functionalized QCM gas sensor. Sens. Actuators B Chem. 248, 820–828. 10.1016/j.snb.2016.12.015

[B45] Wang R. Wang L. Callaway Z. T. Lu H. Huang T. J. Li Y. (2017). A nanowell-based QCM aptasensor for rapid and sensitive detection of avian influenza virus. Sens. Actuators B Chem. 240, 934–940. 10.1016/j.snb.2016.09.06723202345

[B46] Xu Y. Lu Y. Zhang P. Wang Y. Zheng Y. Fu L.. (2020). Infrageneric phylogenetics investigation of Chimonanthus based on electroactive compound profiles. Bioelectrochemistry 133:107455. 10.1016/j.bioelechem.2020.107455 31978859

[B47] Xue M. Ji X. Liang H. Liu Y. Wang B. Sun L.. (2018). The effect of fucoidan on intestinal flora and intestinal barrier function in rats with breast cancer. Food Funct. 9, 1214–1223. 10.1039/C7FO01677H 29384543

[B48] Yang D. Wu X. Yu X. He L. Shah N. P. Xu F. (2017). Mutual growth-promoting effect between *Bifidobacterium bifidum* WBBI03 and *Listeria monocytogenes* CMCC 54001. J. Dairy Sci. 100, 3448–3462. 10.3168/jds.2016-11804 28259400

[B49] Zhang D. Wang D. Zong X. Dong G. Zhang Y. (2018). High-performance QCM humidity sensor based on graphene oxide/tin oxide/polyaniline ternary nanocomposite prepared by in-situ oxidative polymerization method. Sens. Actuators B Chem. 262, 531–541. 10.1016/j.snb.2018.02.012

[B50] Zheng Y. Fu L. Han F. Wang A. Cai W. Yu J. (2015). Green biosynthesis and characterization of zinc oxide nanoparticles using Corymbia citriodora leaf extract and their photocatalytic activity. Green Chem. Lett. Rev. 8, 59–63. 10.1080/17518253.2015.1075069

